# The effect of feeding high fat diet to beef cattle on manure composition and gaseous emission from a feedlot pen surface

**DOI:** 10.1186/s40781-016-0104-6

**Published:** 2016-06-10

**Authors:** Dhan Prasad Gautam, Shafiqur Rahman, Md Saidul Borhan, Chanda Engel

**Affiliations:** Department of Agricultural and Biosystems Engineering, North Dakota State University, Fargo, ND 58102 USA; NDSU Carrington Research Extension Center, Carrington, ND 58421 USA

**Keywords:** Manure composition, Gaseous emission, Diet, Volatile fatty acids, Concentration

## Abstract

**Background:**

Dietary manipulation is a common practice to mitigate gaseous emission from livestock production facilities, and the variation of fat level in the diet has shown great influence on ruminal volatile fatty acids (VFA) and enteric methane generation. The changes in dietary fat levels influence rumen chemistry that could modify manure nutrient composition along with odor and gaseous emissions from manure management facilities.

**Methods:**

A field experiment was carried out on beef cattle feedlots to investigate the effect of four levels of dietary fat concentrations (3 to 5.5 %) on the manure composition and gaseous emissions (methane-CH_4_, nitrous oxide-N_2_O, carbon dioxide-CO_2_ and hydrogen sulfide-H_2_S) from the feedlot pen surface. The experiment was carried out over a 5-month period from June to October during North Dakota’s summer-fall climatic condition. Air and manure sampling was conducted five times at a 20–30 day intervals.

**Results:**

Overall, this research indicated that fat levels in diet have no or little effect on the nutrient composition of manure and gaseous emission from the pens with cattle fed with different diet. Though significant variation of gaseous emission and manure composition were observed between different sampling periods, no effect of high fat diet was observed on manure composition and gaseous emission.

**Conclusions:**

It can be concluded that addition of fat to animal diet may not have any impact on gaseous emission and manure compositions.

## Background

The United States of America is one of the largest producers of livestock and number one producer of beef cattle in the world [1]. According to the USDA, as of July 2015, there are 98.4 million beef cattle in the United states [2] and approximately 1.5 billion kg of manure (according to ASABE Standard D384.2, manure production from a beef cattle is 20–34 kg of manure per day) is generated daily only from beef cattle. Livestock manure is a nutrient source for crops. At the same time, it is also a major source of pollutant gases (ammonia-NH_3_, hydrogen sulfide-H_2_S, etc.), greenhouse gases (GHGs), volatile organic compounds (VOCs), odor, and particulate material (PM). Emission of pollutant gases and GHGs are becoming an important issue for human and animal health, and environment [3, 4]. In a livestock production systems, the rate and amount of gaseous emissions depend on animal species, diet composition, manure management, weather, types of housing system, and topographic features [5].

In a confined livestock operation, the emission of pollutant gases can impact workers’ health, livestock welfare and productivity. The exposure of pollutant gas like H_2_S can cause dizziness, headache, respiratory problem, bronchitis, pulmonary paralysis, and unconsciousness and higher concentration (>100 ppm) can have lethal outcomes [6–8]. Similarly, the concentration more than 25 ppm of NH_3_ can cause respiratory irritation, chemical burns to the respiratory track, skin and eyes, severe cough, and chronic lung diseases [9]. Besides the impacts on human and animal health; those pollutant gases have an impact on environment. For example, NH_3_ can contribute to nutrient build up and eutrophication of surface water, acidification, and the promotion of bacterial growth that leads to weathering and corrosive damage of buildings [10–12]. Livestock production systems generate GHGs and are likely to contribute to the global warming [13, 14].

The GHGs have the potential to absorb and emit infrared radiation that increases the earth’s temperature and cause global warming [15]. The principal GHGs are water vapor, ozone (O_3_), carbon dioxide (CO_2_), methane (CH_4_) nitrous oxide (N_2_O), chlorofluorocarbon, per-fluorocarbon and sulfur hexafluoride; however CH_4_, N_2_O and CO_2_ are the major GHGs emitted from livestock production systems [13, 14]. It is estimated that 3.4 % of the total GHGs emissions in the USA is emitted from livestock [16]. In general, methane is emitted mostly from cattle production systems due to enteric fermentation in rumen and decomposition of manure in the manure treatment and management facilities. Similarly, N_2_O is produced during alternate aerobic and anaerobic decomposition of livestock manure [1]. Though the reported contribution of CH_4_ and N_2_O are only around 9.5 and 5.3 %, respectively, to the total GHG emissions [17]; the global warming potential of these gases are 25 and 298 times of CO_2_, respectively [17]. On the other hand, CH_4_ and N_2_O emission from livestock manure management has increased by 68 and 25 %, respectively, since 1990 [18]. Researchers around the world are seeking technologies and management practices to mitigate emission of these gases from livestock production facilities [19–21]. Among treatment options, diet manipulation is one of the prominent options for minimizing the total gaseous emission (enteric and from manure management) [22–24].

Manure management is one of the major sources of CH_4_ and N_2_O emission; however, a larger portion of CH_4_ (25.9 % of total CH_4_ emission) is also emitted during enteric fermentation in rumens [17]. Basically, the enteric CH_4_ production in rumen is affected by cattle feeding practices and feed diet composition [25]. Specifically, the diet composition can affect rumen pH, carbon nitrogen ratio, nutrient composition of manure, odor, and gaseous emissions from the manure system [6, 26]. In ruminal animal diets, carbohydrate and amount of intake influence the production of individual volatile fatty acids (VFAs), which is directly related with CH_4_ production. Diet with higher sugar and starch components favor propionic acid production resulting in less CH_4_ production [27]. Carbohydrate has the greatest impact on pH, microbial population, and VFA concentration which influences CH_4_ production. Similarly, an increase of fat levels in cattle diets increases the energy density of the diet (8.8 kcal g^-1^) [28], and also help to decrease enteric CH_4_ production [29].

The addition of supplemental fat in the cattle diets is one of the management practices adopted by farmers [30]. The fat content of commercial beef cattle feed is typically 2–5 % [31]. If the fat content in feed exceeds 6 %, it can cause digestive disturbance, diarrhea, and reduce feed intake [32]. Many researchers have conducted experiments using fat and oil in beef cattle diets and observed its impact on body performance, weight gain, cold tolerance, and gaseous emission from body and manure. Engstrom et al. [33] conducted a feeding trial on feedlot performance and carcass quality with beef cattle in Canada using 0, 2 and 4 % fat from canola oil in diets. They found an increase of 9.8 % in daily weight gain with the addition of 4 % fat in diet during the first 56 days.

The increase of fat level in the diet may affect metabolic changes in the ruminant. It may favor the production of propionic acid, which can reduce CH_4_ generation. In addition, supplementary fat can also lower the digestibility of fermentable substrates in the rumen, bio-hydrogenate unsaturated fat, and decrease methanogens population in rumen; ultimately reducing CH_4_ emission [27]. Mathison [34] reported 33 % reduction in enteric CH_4_ production is achievable by adding 4 % canola oil in a steer diet containing 85 % concentrate. Beauchemin and McGinn [35] carried out an experiment using fumeric acid, essential oil, and canola oil in beef cattle diets to observe their effect on enteric CH_4_ emission. Their results showed a reduction on CH_4_ emission using canola oil; though essential oil and fumeric acid did not influence ruminal fermentation or CH_4_ emission. Similarly, Beauchemin et al. [36] used the fat sources from different oil seeds like sunflower, canola and flaxseed to feed the cattle, and observed significant CH_4_ reduction in all cases.

Corn based distiller’s dried grain with solubles (DDGS) is a by-product from the ethanol industries and widely used in livestock diets. Usually, DDGS contains 12 to 15 % oil on a dry basis; however, partial removal of corn oil is common in the ethanol industry. Typically, 3 to 9 % corn oil levels are found in the commercially available DDGS feedstuffs [37]. In beef cattle diets, DDGS is a major ingredient comprising up to 42 % of the total diets [38]. Besides DDGS; corn grain, corn silage, hay, sunflower meal, and concentrated separator by-product (CSB) are some other common ingredients added to beef cattle diets. The desired fat levels in the diets can be achieved by adjusting the inclusion level of DDGS in the diets. However, to the best of our knowledge no studies have been reported on the effect of various fat levels from DDGS on gaseous emission and manure composition from the feedlot pen surfaces. Therefore, the objective of this study was to investigate the effect of different fat levels in beef cattle diets on manure nutrient composition and GHG emission from feedlot pen surfaces.

## Methods

The experimental design and procedures of this study were reviewed and approved by the North Dakota State University Institutional Animal Care and Use Committee (protocol number A13068).

### Feedlot description and experimental design

The research was carried out in a research feedlot at the North Dakota State University Carrington Research Extension Center (CREC). The feedlot had 16 pens and each pen with an area of 433 m^2^ (≈19 m × 23 m). The overall slope of the feedlot was around 3 %. A total of 182 fall-born (*n* = 92) and spring-born (*n* = 90) Angus-steer calves in a randomized block design. Steers were blocked by weight (four groups: light, medium light, medium heavy and heavy). After blocking, the steers were allocated to one of 16 pens (11 to 12 steers per pen) and pen was allocated to 1 of 4 dietary fat levels treatment diets (high fat; medium fat, low fat, and control). Initially, the finishing ration was provided to the heavy and medium heavy animals while the growing ration was provided to light and medium light animals. However, after June the same ration (finishing) was provided to all. This study was conducted from June to October of 2013. The information about animal number, blocking groups, feeding strategies, treatment category and weight of animals on each pen on different time period has been provided in Table 1.

**Table 1:** Summary of animal weight, feeding stage, treatments base on fat levels in diet, and animal weight at different period

Pen#	Animal weight	Feeding stage	Treatments	# of Animals	Weight of animals (kg) (Average ± Standard Deviation)				
					7 June	17 July	14 August	11 Sept.	2 Oct.
Pen 1	Heavy	Finish	Medium fat	11	448 ± 16	528 ± 23	588 ± 37	642 ± 43	683 ± 48
Pen 2	Heavy	Finish	Low fat	12	451 ± 19	533 ± 33	595 ± 39	654 ± 48	697 ± 40
Pen 3	Heavy	Finish	High fat	11	448 ± 17	535 ± 21	608 ± 21	671 ± 26	715 ± 31
Pen 4	Heavy	Finish	Control	11	446 ± 13	529 ± 18	586 ± 19	651 ± 20	695 ± 26
Pen 5	Medium- heavy	Finish	Medium fat	11	411 ± 12	504 ± 23	569 ± 28	632 ± 33	677 ± 40
Pen 6	Medium- heavy	Finish	Control	11	413 ± 12	494 ± 24	559 ± 34	619 ± 35	661 ± 38
Pen7	Medium- heavy	Finish	High fat	11	412 ± 13	490 ± 14	556 ± 20	622 ± 27	661 ± 25
Pen 8	Medium- heavy	Finish	Low fat	12	413 ± 13	489 ± 23	548 ± 27	613 ± 33	655 ± 39
Pen 9	Medium-light	Growing/Finish	Medium fat	11	358 ± 20	426 ± 23	491 ± 21	552 ± 29	628 ± 33
Pem10	Medium-light	Growing/Finish	Low fat	11	358 ± 18	426 ± 26	487 ± 29	545 ± 31	629 ± 38
Pen 11	Medium-light	Growing/Finish	High fat	12	360 ± 21	433 ± 25	508 ± 31	572 ± 31	649 ± 34
Pen 12	Medium-light	Growing/Finish	Control	12	360 ± 21	429 ± 22	500 ± 34	554 ± 33	638 ± 39
Pen 13	Light	Growing/Finish	Medium fat	11	307 ± 18	380 ± 26	441 ± 34	505 ± 43	585 ± 47
Pen 14	Light	Growing/Finish	High fat	12	307 ± 19	384 ± 17	455 ± 22	516 ± 22	596 ± 34
Pen 15	Light	Growing/Finish	Control	11	306 ± 20	383 ± 24	448 ± 23	505 ± 28	594 ± 29
Pen 16	Light	Growing/Finish	Low fat	12	309 ± 18	386 ± 23	443 ± 29	506 ± 32	588 ± 36

### Weather condition

During each sampling, the pen surface temperatures were measured using an infrared thermometer (MiniTemp-MT6 Instrument, Carlsbad, CA). Ambient temperature, wind speed, solar radiation, and rainfall were collected from the North Dakota Agricultural Weather Network - NDAWN site, NDSU Carrington Research and Extension center, located 2 km from the study site.

### Dietary composition

In this study, the effects of four different dietary fat levels (high, medium, low and control) on beef cattle performance, manure composition and gaseous emissions from feedlot pen surfaces were studied. Three different DDGS products sourced from different ethanol plants, were used to obtain different oil levels. High fat treatment group used DDGS purchased from High-water Ethanol, Lamberton, MN; and had 12.96 % corn oil (no corn removal). Medium fat treatment group consisted of DDGS purchased from Blue Flient Ethanol, Washburn, ND; which used 8.05 % corn oil (partial removal). Similarly, low fat treatment group consisted of DDGS purchased from POET, Groton, SD; used 5.47 % corn oil (higher removal). The control diet included sunflower meal used 2.44 % oil. Besides DDGS, other ration ingredients were chopped grass hay, dry-rolled corn grain, corn silage, condensed separator by product and a vitamins and minerals supplements. The diets were formulated to meet the nutrient requirement recommended by NRC [39]. Overall, the fat content of high, medium, low, and control diet (composite diet) were 5.07, 4.12, 3.6, and 3.19 %, respectively in the growing ration and they were 5.48, 4.52, 4.02, and 3.58 %, respectively in the finishing ration. The diet ingredients and the nutrient composition of composite diet is listed in Table 2, and the nutrient composition of each ingredient is listed in Table 3.

**Table 2:** Diet ingredient and nutrient composition of growing and finishing ration

Diet ingredients	Growing Rations				Finishing Rations			
	Control	High Fat	Med. Fat	Low Fat	Control	High Fat	Med. Fat	Low Fat
Corn (%)	48.76	43.25	42.76	43.09	66.68	61.02	60.95	60.96
DDGS (%)	--	18.89	18.76	18.7	--	19.4	19.34	19.4
Sunflower meal (%)	13.24	--	--	--	13.3	--	--	--
Hay (%)	16.96	16.95	17.06	16.96	11.13	11.27	11.29	11.29
Corn silage (%)	12.85	12.9	13.18	13.04	--	--	--	--
CSB (%)	6.32	6.3	6.27	6.29	6.76	6.73	6.73	6.73
Supplement (%)	1.87	1.71	1.96	1.92	1.56	1.58	1.69	1.62
Nutrient Composition								
CP (%)	12.09	11.85	12.39	12.53	12.42	12.12	12.7	12.88
NEm (Mcal kg^-1^)	0.37	0.37	0.37	0.37	0.37	0.41	0.41	0.41
NEg (Mcal kg^-1^)	0.23	0.23	0.23	0.23	0.24	0.27	0.27	0.27
Fat (%)	3.19	5.07	4.12	3.64	3.58	5.48	4.52	4.02

Note: DDGS = Distiller’s dried grains with solubles; CSB = Concentrated separator by-product; CP = Crude protein; NEm = Net energy of maintenance; NEg = net energy of gain; All feed samples were analyzed by a commercial laboratory; net energy prediction calculations were done as per NRC [58–60]

**Table 3:** Nutrient composition in each diet ingredient

Ingredient	DM %	CP %	ADF %	TDN %	NEm Mcal kg^-1^	NEg Mcal kg^-1^	Fat %
Corn	87.66	8.33	3.58	87	0.45	0.31	4.37
Corn silage	32.90	7.53	28.69	68	0.33	0.20	2.59
Mixed hay	85.74	7.42	44.70	52	0.22	0.10	1.88
Sunflower meal	90.63	39.44	22.77	70	0.34	0.21	2.44
DDGS medium fat	89.26	31.90	16.48	87	0.44	0.29	8.05
DDGS high fat	88.74	28.76	15.74	88	0.44	0.29	12.96
DDGS low Fat	88.83	32.69	11.93	92	0.43	0.29	5.47
CSB	71.56	10.07	0.19	86	0.42	0.28	1.28

Note: *DDGS* Distiller’s dried grains with solubles, *CSB* Concentrated separator by-product, *DM* dry matter, *CP* Crude protein, *ADF* Acid detergent fiber, *TDN* Total digestible nutrients, *NEm* Net energy for maintenance, *NEg* Net energy for gain. All feed samples were analyzed by a commercial laboratory; net energy prediction calculations are from [58–60]

### Gaseous sampling and analysis

**Fig. 1:**
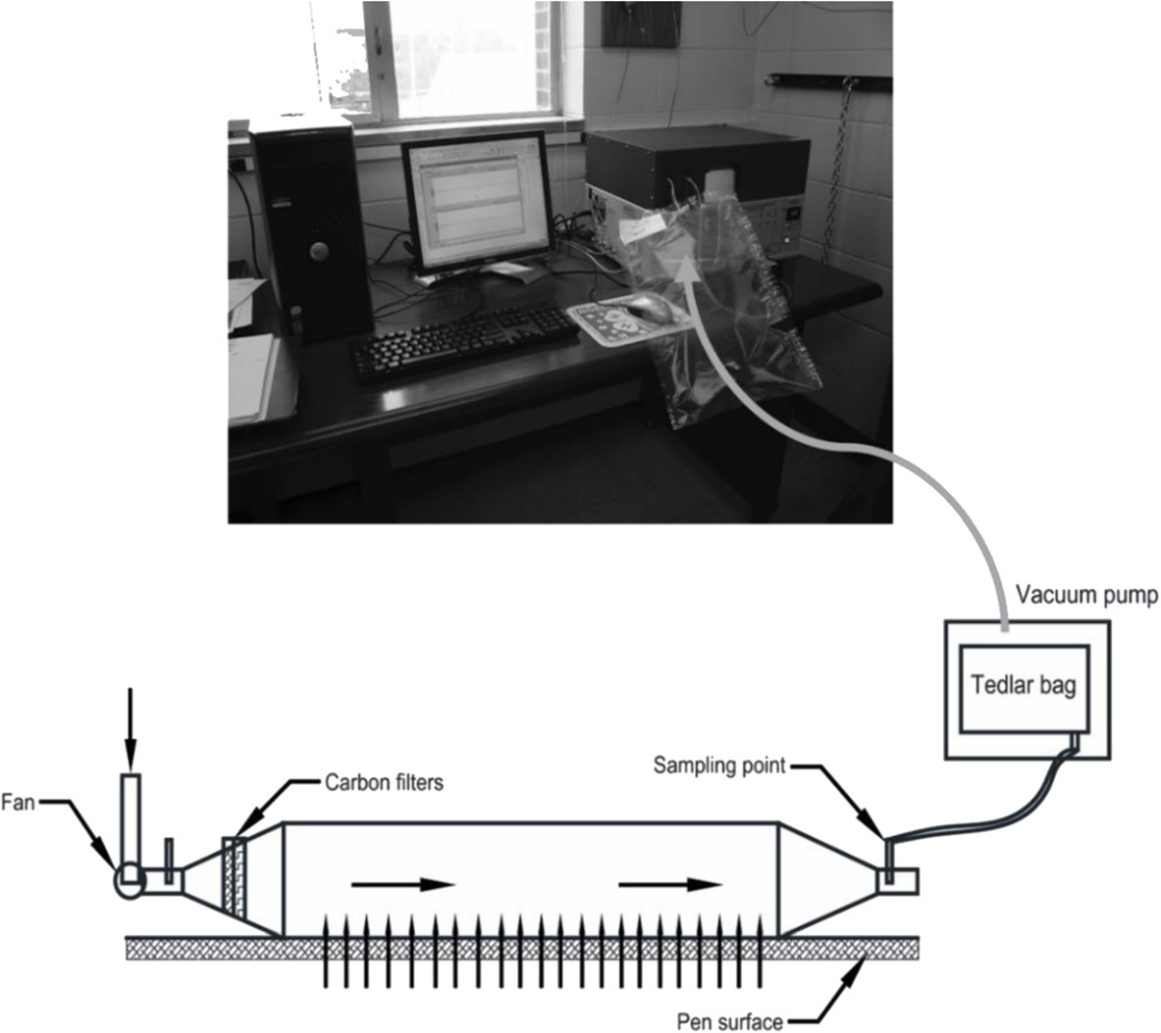
Schematic diagram of gas collection using a wind tunnel and GHG analysis using a gas chromatograph (drawing not to scale)

Air samples from the pen surface were collected for five times during June to October 2013 with a sampling interval of 30 ± 10 days. Air samples were collected using a custom built portable wind tunnel with a foot print area of 0.32 m^2^ (0.8 m × 0.4 m), Tedlar bag, and Vac-U-Chamber (SKC Inc., Eighty Four, PA) (Fig. 1). Measured air velocity over the foot-print area of tunnel immediate over the manure surface was maintained 0.35 m s^-1^ that prompted an air flow through the tunnel of 2.75 L s^-1^ (0.00275 m^3^ s^-1^). In each sampling location, a 5 L Tedlar bag was placed inside a vacuum chamber and a uniform air flow rate (2.75 L s^-1^) was maintained inside the tunnel throughout the sampling period using a DC motor. Additional sampling protocol can be found at Rahman et al. [40]. In each pen, two samples were collected; one from the front end of the pen next to feeding area, and another one from the backside of pen. So, a total of 160 air samples (16 pens × 2 samples/pen × 5 times) were collected and they were brought back to the laboratory for H_2_S, CH_4_, CO_2_, and N_2_O analysis.

Within 24 h of sampling, they were analyzed for GHGs concentration using a greenhouse gas GC (Model No. 8610C, SRI Instruments, and 20720 Earl St., Torrance, CA 90502), and H_2_S concentration using a Jerome meter (Jerome^®^ 631-X, Arizona instrument, Arizona, USA). The GC was equipped with a flame ionization detector (FID) to measure CO_2_ and CH_4_ and an electron captured detector (ECD) to analyze N_2_O. GHG was analyzed following the procedure described in Rahman et al. [40].

### Manure sampling and analysis

Manures on the pen surface were allowed to accumulate until animals were sold out. During a sampling event, the manure sampling was paired with air sampling and relatively fresh manures (freshly excreted or few minutes old) were sampled. The composite manure samples collected from five to seven spots in a pen, bagged and mix in a zip-locked bag, kept in ice cooled cooler in the field and during transportation; and finally stored in a refrigerator at around 4 °C until analysis. Thus, in each sampling day, a total of 16 composite manure (each approximately ~ 800 g) samples were collected. Before analysis, samples were mixed thoroughly again, divided into two sub-samples (sub-sample 1: pH, total nitrogen (TN), potassium (K), total phosphorus (TP) and total carbon (TC); sub-sample 2: volatile fatty acids (VFAs), crude protein (CP) and ammonical nitrogen), and were sent to two analytical laboratory for analysis following the standard laboratory methods (Table 4).

**Table 4:** Method/protocol used to analyze manure samples

Parameters	Methods/protocol used
TN	Recommended methods of manure analysis, A3769 Macro-Kjeldahl method (adapted from Kane, 1998)
K	Recommended method of manure analysis, A3769
TP	Recommended method of manure analysis, A3769
TC	U.S. EPA method 415.1: Catalytic combustion and non-dispersive infrared detection (NDIR) method
CP	Official Method 2001.11, AOAC International (2005) 18^th^ ED., AOAC^1^ International Gaithersburg, MD, USA
NH_3_-N	Sigma Technical Bulletin #640. Sigma Diagnostics, St. Louis, MO 63178
VFA	Method of Goetsch and Galyean, 1983. Agilent 6890 N Gas Chromatograph with a FID (flame ionization detector) and the 7683 Series auto injector and auto sampler. Column used was the Supelco brand, NUKOL Fused Silica Column, 15 m × 0.53 mm × 0.5 um

^1^ *AOAC* Association of Official Agricultural Chemists

### Emission calculation

In order to estimate the emission rate; the volumetric gas concentration was standardized at standard pressure and temperature (1 atm and 25 °C), and mass concentration of the compound was calculated from calculated volumetric concentration (Equation 1). Flux rates (g m^-2^ d^-1^) was calculated using the average airflow through the wind tunnel, mass concentration of the target gas and the surface area covered by the wind tunnel as shown in Equation 2. Finally, emission rate was estimated using the surface area of pen, flux rate, and animal unit (AU) in the pen (Equation 3).

1 $$ {C}_{mass}=\frac{C_{ppm}\times MW\ }{24.24} $$

where, C_ppm_ = Volumetric concentration of the target gas (ppm)

C_mass_ = Mass concentration of the target gas (mg m^-3^)

MW = Molecular weight of the target gas (g mol^-1^)

24.25 = Volume per mole of an ideal gas at standard temperature and pressure (L mol^-1^)

2 $$ FR=\frac{C_{mass}\times {V}_{wt}\times 3600\times 24}{A_{wt}\times 1000} $$

where, FR = GHG emission flux rate from pen surface (g m^-2^ d^-1^)

V_wt_ = Airflow rate through wind tunnel (m^3^ s^-1^)

A_wt_ = Surface area covered by the wind tunnel (0.4 × 0.8 m^2^)

3 $$ ER=\frac{FR\times {A}_{Sc}}{AU} $$

where, ER = GHG emission rate from pen surface (g hd^-1^ d^-1^)

A_sc_ = Surface area of the source (m^2^)

AU = Animal unit (total weight of animals in pen divided by 500 kg live weight)

### Ambient weather and feedlot pen surface temperature measurement

**Fig. 2:**
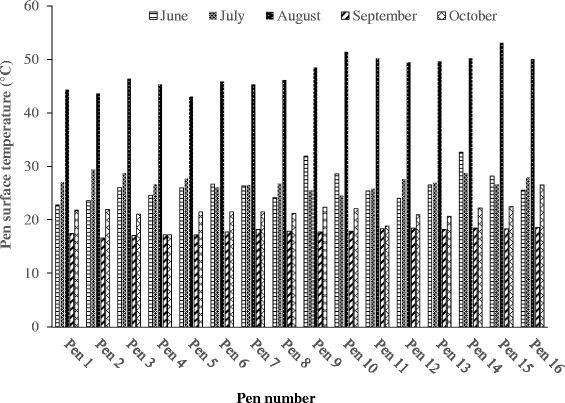
Variation in feedlot pen surface temperature during the experimental period

The daily mean air temperature, wind speed, solar irradiation, and rainfall at the sampling locations during each sampling period are listed in Table 5. The August sampling time had the highest ambient temperature, while October had the lowest ambient temperature. Likewise, the highest pen surface temperature was noted in August, which equates to the ambient temperature (Fig. 2). Similarly, the lowest pen surface temperature was observed in September. Overall, average pen surface temperatures were very consistent among pens at each sampling time. Besides temperature, solar radiation was also the highest in August, and the lowest in September. During the sampling time, no noticeable rainfall was observed, which might have some effects on gaseous emission from the manure pen surface.

**Table 5:** Ambient weather condition at the study site

Sampling date	Air temperature (°C)			Average wind speed (m s^-1^)	Solar radiation (MJ m^-2^)	Rainfall (mm)
	Average	Minimum	Maximum			
20-Jun-13	20.56	17.78	23.33	5.09	7.57	0.00
30-July-13	16.67	10.56	22.78	2.14	16.99	0.80
20-Aug-13	26.11	16.11	36.11	2.77	23.01	0.00
18-Sep-13	18.33	13.89	22.78	2.55	5.19	0.00
9-Oct-13	11.11	2.78	19.44	1.52	10.08	0.00

### Statistical analysis

It is known that temperature variation on the pen surface effects gaseous emissions. Usually, higher temperatures enhance CH_4_ production [41]. The temperature range of 25–30 °C is considered optimum for CH_4_ production [42]. Surface temperature also influence on N_2_O emission. Luo et al. [43] reported the highest N_2_O emissions with moist and warm soil, and the soil exposed to freezing and thawing condition. Lang et al. [44] also observed the higher soil temperature promoted greater nitrification and N_2_O emissions.

The effect of fat levels in the diet on GHG emission and manure composition were compared using the Generalized Liner Model (GLM) procedure in SAS software (SAS 9.3, 2002-2010). Randomized complete block design was chosen for each sampling event (months) with animal weight as a block (light and heavy) for four treatments (control, low, medium and high). However, during analysis no significant difference of treatments were observed separating the animals on weight basis. Therefore, a comparative study of different treatments were carried considering the animal types as a single block. All significance tests were evaluated at *P* = 0.05. The null hypothesis was that the means value of GHGs concentrations, emission flux, emission rates, manure nutrient and VFAs concentrations were equal within and among treatments and sampling time.

## Results and discussion

### Manure composition

Overall, no significant differences in manure composition were observed among treatments in most of the sampled months, but significant differences on some manure composition were observed over the sampling period (Table 6). Likewise in August, the moisture content, crude protein (CP), and TN were also significantly lower in manure from pens with cattle fed high fat diets compared to other treatment groups.

**Table 6:** Average manure composition for each month based on treatment

Sampling date	Manure composition	Treatments			
		control	Low fat	Medium fat	High fat
20-Jun-13	pH	5.4 a^*^ ± .0.2	5.6 a ± 0.4	5.5 a ± 0.4	5.4 a ± 0.2
	Moisture % wb	76.7 a ±1.9	76.9 a ± 1.0	74.2 a ± 5.5	77.4 a ± 1.6
	Ash %	11.1 a ± 1.4	13.9 a ± 4.2	10.0 a ± 0.8	10.4 a ± 1.6
	CP %	14.9 a ± 0.8	14.9 a ± 1.4	13.7 a ± 2.8	15.1 a ± 0.6
	TN %	2.4 a ± 0.1	2.4 a ± 0.2	2.2 a ± 0.5	2.4 a ± 0.1
	NH_3_ (mM)	5.6 a ± 1.2	4.9 a ± 1.7	5.5 a ± 1.6	4.7 a ± 1.4
	TC (%)	43.7 a ± 1.0	43.2 a ± 1.5	44.8 a ± 0.5	43.7 a ± 1.0
	TP %	0.1 a ±0.1	0.2 a ± 0.1	0.2 a ± 0.1	0.1 a ± 0.0
	K %	0.1 a ± 0.0	0.1 a ± 0.0	0.1 a ± 0.0	0.1 a ± 0.0
30-Jul-13	pH	5.5 a ± 0.1	5.4 a ± .2	5.5 a ± .4	5.5 a ± 0.1
	Moisture % wb	77.8 a ± 0.6	77.2 a ± 1.5	76.8 a ± 1.5	75.5 a ± 2.6
	Ash %	9.1 a ± 0.8	8.2 a ± 0.3	10.2 a ± 1.3	8.5 a ± 1.6
	CP %	15.6 a ± 1.5	15.4 a ± 1.1	16.3 a ± 0.8	14.9 a ± 1.0
	TN %	2.5 a ± 0.3	2.5 a ± 0.2	2.6 a ± 0.1	2.4 a ± 0.1
	NH_3_ (mM)	5.2 a ± 1.7	5.5 a ± 1.5	9.4 a ± 5.7	8.9 a ± 2.1
	TC (%)	44.4 a ± 0.4	43.6 a ± 1.8	44.3 a ± 0.4	45.4 a ± 0.8
	TP %	0.1 a ± 0.0	0.1 a ± 0.0	0.1 a ± 0.0	0.1 a ± 0.0
	K %	0.1 a ± 0.0	0.1 a ± 0.0	0.1 a ± 0.0	0.1 a ± 0.0
20-Aug-13	pH	5.2 a ± 0.1	5.1 a ± 0.3	5.1 a ± 0.1	5.0 a ± 0.2
	Moisture % wb	73.7 ab ± 1.2	73.3 ab ± 2.2	75.6 a ± 0.7	72.2 b ± 1.9
	Ash %	8.1 a ± 0.5	8.9 a ± 1.1	7.7 a ± 0.6	7.4 a ± 1.0
	CP %	15.2 ab ± 1.1	17.6 a ± 1.4	17.4 ab ± 1.8	14.9 b ± 0.6
	TN %	2.4 ab ± 0.2	2.8 a ± 0.2	2.8 ab ± 0.3	2.4 b ± 0.1
	NH_3_ (mM)	12.8 a ± 1.2	17.1 a ± 9.0	16.8 a ± 6.8	11.3 a ± 3.7
	TC (%)	44.2 a ± 1.3	45.0 a ± 2.2	44.3 a ± 0.5	42.9 a ± 1.8
	TP %	0.1 a ± 0.0	0.1 a ± 0.1	0.1 a ± 0.0	0.2 a ± 0.0
	K %	0.1 a ± 0.0	0.1 a ± 0.0	0.1 a ± 0.0	0.1 a ± 0.0
18-Sep-13	pH	5.6 a ± 0.2	5.4 a ± 0.2	5.4 a ± 0.3	5.5 a ± 0.1
	Moisture % wb	75.4 a ± 1.4	74.9 a ± 2.0	75.1 a ± 2.8	75.7 a ± 1.3
	Ash %	9.5 a ± 2.1	8.0 a ± 1.3	8.7 a ± 1.0	7.9 a ± 1.0
	CP %	15.0 a ± 1.5	15.0 a ± 2.6	15.6 a ± 0.8	15.0 a ± 1.1
	TNm %	2.4 a ± 0.2	2.4 a ± 0.4	2.5 a ± 0.1	2.4 a ± 0.2
	NH_3_ (mM)	10.0 ab ± 2.9	7.6 b ± 3.3	8.0 ab ± 2.1	11.5 a ± 2.3
	TC (%)	44.4 a ± 0.8	44.1 a ± 0.7	43.5 a ± 1.7	44.9 a ± 0.4
	TP %	0.2 a ±0.1	0.1 b ± 0.0	0.1 b ± 0.0	0.1 b ± 0.0
	K %	0.1 a ± 0.0	0.1 a ± 0.0	0.1 a ± 0.0	0.1 a ± 0.1
9-Oct-13	pH	5.5 a ± .01	5.6 a ± 0.1	5.9 a ± .02	5.3 a ± 0.0
	Moisture % wb	72.6 a ± 0.5	72.9 a ± 0.0	72.4 a ± 3.6	72.4 a ± 3.0
	DM %	95.6 a ± 0.2	96.7 a ± 0.0	96.6 a ± 0.5	96.1 a ± 0.3
	Ash %	8.7 a ± 1.3	8.5 a ± 1.2	19.9 a ± 10.9	6.9 a ± 0.9
	CP %	16.4 a ± 1.2	14.9 a ± 1.2	13.9 a ± 1.1	15.0 a ± 0.6
	TN %	2.6 a ± 0.2	2.4 a ± 0.2	2.2 a ± 0.2	2.4 a ± 0.1
	NH_3_ (mM)	6.9 a ± 0.9	14.9 a ± 2.7	19.0 a ± 5.7	8.0 a ± 1.1
	TC (%)	44.0 a ± 0.9	39.9 a ± 5.0	37.2 a ± 7.2	44.8 a ± 0.2
	TP %	0.1 a ± 0.0	0.2 a ± 0.1	0.1 a ± 0.0	0.1 a ± 0.0
	K %	0.1 a ± 0.0	0.2 a ± 0.0	0.2 a ± 0.0	0.1 a ± 0.0

^*^Values followed by the same letter in row are not significantly different at *P* ≤ 0.05; wb = wet basis

However, when the analysis was simply carried out on time basis (comparison among months), significant difference on most of the parameters of manure composition were observed (Table 7). Manure pH was significantly lower in August as compared to other months. Similarly, moisture content of manure was significantly lower in October as compared to June, July and September as shown in Table 7. Ash content of manure was the highest in June and the lowest in August. Crude protein, TP, and ammonical nitrogen NH_3_-N content in manure were the lowest in June and the highest in August. Total carbon (TC) in manure was significantly lower in August as compared to other months. In 2012 summer, Borhan et al. [45] had also measured the nutrient composition of the manure in the same feed lot under similar condition and the values of nutrient parameter were almost comparable with this study.

**Table 7:** Average manure composition on monthly basis

Parameters	June	July	August	September	October
pH	5.5 a^*^ ± 0.1	5.5 a ± 0.0	5.1 b ± 0.1	5.5 a ± 0.1	5.6 a ± 0.2
Moisture %	76.32 a ± 1.2	76.8 a ± 0.8	73.7 bc ± 1.2	75.3 ab ± 0.3	72.6 c ± 0.2
Ash %	11.3 a ± 1.5	9.0 abc ± 0.8	8.0 c ± 0.6	8.5 bc ± 0.5	11.0 ab ± 5.2
CP %	14.6 a ± 0.6	15.5 ab ± 0.5	16.2 b ± 1.2	15.1 ab ± 0.3	15.0 ab ± 0.9
TN %	2.3 a ± 0.1	2.5 ab ± 0.1	2.6 b ± 0.2	2.4 ab ± 0.0	2.4 ab ± 0.1
NH_3_-N (mM)	5.2 d ± 0.4	7.3 cd ± 1.9	14.5 a ± 2.5	9.3 bc ± 1.6	12.2 ab ± 5.0
TC (%)	43.8 a ± 0.6	44.4 a ± 0.6	44.1 a ± 0.8	44.2 a ± 0.5	41.5 b ± 3.1
TP %	0.1 a ± 0.0	0.1 a ± 0.0	0.1 a ± 0.0	0.2 a ± 0.0	0.1 a ± 0.0
K %	0.1 a ± 0.0	0.1 a ± 0.0	0.1 a ± 0.0	0.1 a ± 0.0	0.1 a ± 0.0

^*^ Values followed by the same letter in row are not significantly different at *P* ≤ 0.05; *CP* Crude protein, *TN* Total nitrogen, *NH*_*3*_ *-N* ammonical nitrogen, *TP* Total phosphorus, *K* Potassium, *TVFA* Total volatile fatty acids, *DM* Dry matter

### Effect of dietary fat level on volatile fatty acid (VFAs) composition of manure

No significant differences in any of VFAs concentration were observed among treatments during the study period except for two months. In July, isovaleric acid was significantly higher in manure from pens with cattle fed the low fat diets than the control. Likewise, in September butyric acid was significantly higher in the manure from pens with cattle fed the medium fat diets compared to the control (Table 8). Similarly in August and September, the total volatile fatty acid (TVFA) content were significantly lower in the high fat group than the others (Table 8), which may contribute to lower CH_4_ emission. However, when the analysis carried out on monthly basis, the lowest acetic acid concentration and the highest propionic acid concentrations were observed in August (Table 9). Likewise, the TVFA content of manure was significantly higher in July and August compared to other months (Table 9), which is likely due to temperature effect on VFA production. Due to higher TVFA, comparatively higher CH_4_ emission can be expected during July and August.

**Table 8:** Volatile fatty acids content in manure measured based on the treatment

Sampling date	VFAs (mM)	Treatments			
		control	Low fat	Medium fat	High fat
20-Jun-13	Acetic	49.5 a^*^ ± 3.2	51.3 a ± 3.5	46.0 a ± 1.5	46.5 a ± 4.8
	Propionic	17.0 a ±2.1	17.3 a ± 2.5	20.2 a ± 4.1	19.4 a ± 4.0
	Isobutyric	2.1 a ± 1.3	2.0 a ± 0.6	2.0 a ± 1.4	2.1 a ± 0.6
	Butyric	24.0 a ± 2.6	21.7 a ± 3.0	25.7 a ± 3.7	23.0 a ± 1.5
	Isovaleric	4.6 a ± 1.4	3.2 a ± 1.9	4.6 a ± 1.7	3.9 a ± 1.3
	Valeric	2.8 a ± 3.0	4.5 a ± 1.3	1.5 a ± 1.5	5.1 a ± 1.8
	TVFA (mM)	120.7 a ± 27.6	101.7 a ± 37.4	105.1 a ± 8.6	127.1 a ± 24.2
30-Jul-13	Acetic	51.4 a ± 2.2	49.5 a ± 2.4	51.1 a ± 2.9	50.9 a ± 2.8
	Propionic	23.5 a ± 1.4	19.8 a ± 3.4	21.3 a ± 3.3	19.5 a ±2.3
	Isobutyric	1.4 a ± 0.3	1.5 a ± 0.4	1.7 a ± 0.6	1.7 a ± 0.2
	Butyric	21.0 a ± 1.2	24.4 a ± 2.3	22.1 a ± 1.8	22.8 a ± 1.7
	Isovaleric	1.3 b ± 0.3	3.5 a ± 0.5	2.2 ab ± 0.8	3.4 ab ± 0.3
	Valaric	1.4 a ± 1.3	1.4 a ± 1.0	1.6 a ± 1.7	1.6 a ± 0.9
	TVFA (mM)	150.2 a ± 18.9	149.0 a ± 32.7	176.4 a ± 42.2	148.7 a ±10.9
20-Aug-13	Acetic	46.0 a ± 3.7	44.7 a ± 2.8	45.6 a ± 2.4	49.0 a ± 5.6
	Propionic	25.4 a ± 1.3	25.6 a ± 3.9	24.7 a ± 2.1	22.0 a ± 3.5
	Isobutyric	1.5 a ± 0.2	1.4 a ± 0.3	1.2 ab ± 0.4	0.7 b ± 3.6
	Butyric	22.4 a ± 2.4	22.4 a ± 2.4	23.4 a ± 1.4	24.6 a ± 3.6
	Isovaleric	1.3 a ± 0.1	1.5 a ± 0.6	1.3 a ± 0.4	0.9 a ± 0.3
	Valeric	3.5 a ± 0.5	4.4 a ± 1.7	1.3 a ± 1.2	2.9 a ± 1.1
	TVFA (mM)	147.6 ab ± 34.8	178.2 a ± 19.9	142.0 ab ± 42.5	128.8 b ± 47.5
18-Sep-13	Acetic	48.9 b ± 0.6	50.8 ab ± 0.6	53.5 a ± 2.1	51.2 ab ± 2.5
	Propionic	22.7 a ± 1.7	22.4 a ± 1.1	20.9 a ± 2.3	21.9 a ± 1.0
	Isobutyric	1.1 a ± 0.3	0.8 a ± 0.1	1.0 a ± 0.5	1.2 a ± 0.3
	Butyric	23.7 a ± 1.0	22.4 ab ± 1.8	20.6 b ± 1.6	21.3 ab ± 1.0
	Isovaleric	1.0 a ± 0.3	0.8 a ± 0.2	1.0 a ± 0.5	1.2 a ± 0.2
	Valeric	2.5 a ± 0.6	2.8 a ± 1.0	3.0 a ± 1.3	3.6 a ± 1.2
	TVFA (mM)	129.2 a ± 15.0	109.0 ab ± 14.4	114.5 ab ± 17.9	105.3 b ± 11.1
9-Oct-13	Acetic	53.5 a ± 0.9	49.8 b ± 0.0	51.9 ab ± 0.3	50.2 b ±0.8
	Propionic	20.9 a ±0.1	20.6 a ± 0.6	20.2 a ± 1.7	22.8 a ± 0.7
	Isobutyric	0.9 a ± 0.0	1.2 a ± 0.0	1.4 a ± 0.4	0.4 a ± 0.5
	Butyric	21.8 a ± 0.2	23.7 a ± 0.9	21.8 a ± 0.5	24.4 a ± 1.3
	Isovaleric	0.9 a ± 0.3	1.2 a ± 0.1	1.4 a ± 0.3	0.6 a ± 0.2
	Valeric	1.9 a ± 0.2	3.5 a ± 1.5	3.3 a ± 1.5	1.5 a ± 1.5
	TVFA (mM)	144.5 a ± 18.4	108.8 a ± 0.5	113.5 a ± 16.2	123.1 a ±8.0

^*^Values followed by the same letter in row are not significantly different at *P* ≤ 0.05

**Table 9:** Monthly volatile fatty acid (VFA) analysis of manure

Parameters	June	July	August	September	October
Acetic	48.3 bc^*^ ± 2.2	50.7 ab ± 0.7	46.3 c ± 1.6	51.1 ab ± 1.6	51.4 a ± 1.5
Propionic	18.5 c ± 1.4	21.0 b ± 1.6	24.4 a ± 1.4	22.0 b ± 0.7	21.1 b ± 1.0
Isobutyric	2.0 a ± 0.1	1.6 ab ± 0.1	1.2 bc ± 0.3	1.0 c ± 0.2	1.0 c ± 0.4
Butyric	23.6 a ± 1.5	22.6 a ± 1.2	23.2 a ± 0.9	22.0 a ± 1.2	22.9 a ±1.1
Isovaleric	4.1 a ± 0.6	1.5 b ± 0.1	1.2 b ± 0.2	1.0 b ± 0.1	1.0 b ± 0.3
Valeric	3.5 a ± 1.4	2.6 a ± 0.9	3.7 a ± 0.6	2.9 a ± 0.2	2.6 a ± 0.8
TVFA (mM)	113.7 b ± 10.6	156.1 a ± 11.8	149.2 a ± 18.1	114.5 b ± 9.1	122.5 b ± 13.7

^*^Values followed by the same letter in row are not significantly different at *P* ≤ 0.05

During anaerobic decomposition of manure; acetic, propionic, butyric and valeric acids are the common VFAs produced by micro-organisms. Acetic acid is the major VFA responsible for CH_4_ production from anaerobic biomass which accounts more than two third of CH_4_ production [46]. Propionic and butyric acids are considered as the inhibitory agents in anaerobic process [47]. Higher concentration of propionic usually inhibits the CH_4_ production in the case of anaerobic digester [48]; however, some researchers have mentioned that it’s the effect rather than cause for the inhibition of CH_4_ production [49, 50]. The ratio of acetic acid and propionic acid is another important factor for determining the CH_4_ production rate. Higher acetic acid (>800 mg L^-1^) as well as propionic acid and acetic acid ratios greater than 1:4 is taken as the indicator for failure of anaerobic processes [51]. However in this study, the ratio of propionic acid to TVFA was <1:4 (Tables 8 and 9), which was an indicator of anaerobic process on the pen surface.

### Effect of dietary fat level on GHG emission

Overall, no significant difference in GHGs emissions were observed from the feedlot pen surfaces with beef cattle fed four levels of fat (control, low, medium, high) in the diet (Table 10). However, some variations on GHG emission were observed when the measurement were compared between months. In July and September, the highest CO_2_ efflux was observed from pen surface with cattle fed medium fat diets. The increased fat source in the diets is likely to increase dietary energy, suppress methanogens decreasing CH_4_ emissions (both enteric and from manure) as well as reduce nitrogen emissions from manure [27, 52]. No significant difference in the total nitrogen and ammonium nitrogen between the treatments also support less variation of N_2_O emission between treatments.

**Table 10:** Analysis of greenhouse gas emissions based on treatment

Sampling date	Manure composition	Treatments			
		control	Low fat	Medium fat	High fat
20-Jun-13	CH_4_ concentration (ppm)	2.2 ab^*^ ± 0.0	2.2 a ± 0.2	2.3 b ± 0.2	2.1 a ± 0.0
	CO_2_ concentration (ppm)	378.0 a ± 24.0	378.3 a ± 17.9	390.0 a ± 40.4	374.3 a ± 15.8
	N_2_O concentration (ppm)	0.9 a ± 0.1	1.2 a ± 0.5	0.8 a ± 0.4	1.4 a ± 0.4
	CH_4_ FR (g m^-2^ d^-1^)	1.1 a ± 0.0	1.1 a ± 0.1	1.1 a ± 0.1	1.0 a ± 0.0
	CO_2_ FR (g m^-2^ d^-1^)	504.8 a ± 32.0	505.2 a ± 23.9	520.8 a ± 54.0	499.8 a ± 21.1
	N_2_O FR (g m^-2^ d^-1^)	1.2 a ± 0.2	1.6 a ± 0.6	1.1 a ± 0.5	1.8 a ± 0.6
	CH_4_ EF (g AU^-1^ d^-1^)	54.3 a ± 8.5	53.4 a ± 9.4	55.7 a ± 6.3	51.4 a ± 6.0
	CO_2_ EF (kg AU^-1^ d^-1^)	25.8 a ± 2.8	24.7 a ± 2.9	27.1 a ± 2.2	25.9 a ± 1.7
	N_2_O EF (g AU^-1^ d^-1^)	62.2 a ± 8 .9	78.2 a ± 26.5	54.5 a ± 20.4	93.8 a ± 35.4
30-Jul-13	CH_4_ concentration (ppm)	2.8 ab ± 0.3	2.8 b ± 0.1	3.1 a ± 0.2	2.6 b ± 0.1
	CO_2_ concentration (ppm)	467.9 b ± 70.3	485.4 b ± 67.0	518.0 a ± 75.0	473.5 b ± 58.8
	N_2_O concentration (ppm)	1.0 a ± 0.4	0.8 a ± 0.2	1.3 a ± 0.1	1.0 a ± 0.3
	CH_4_ FR (g m^-2^ d^-1^)	1.4 ab ± 0.2	1.3 b ± 0.0	1.5 a ± 0.1	1.3 b ± 0.1
	CO_2_ FR (g m^-2^ d^-1^)	624.7 b ± 93.9	648.2 b ± 89.5	691.7 a ± 100.2	632.2 b ± 78.5
	N_2_O FR (g m^-2^ d^-1^)	1.4 a ± 0.5	1.1 a ± 0.3	1.7 a ± 0.1	1.4 a ± 0.4
	CH_4_ EF (g AU^-1^ d^-1^)	58.4 ab ± 5.6	54.8 a ± 6.3	64.3 b ± 4.2	52.8 a ± 3.6
	CO_2_ EF (g AU^-1^ d^-1^)	26.2 a ± 1.6	26.1 a ± 2.9	29.6 b ± 2.2	25.9 a ± 1.7
	N_2_O EF (kg AU^-1^ d^-1^)	58.6 ab ± 23.5	42.5 a ± 7.0	74.1 b ± 9.5	55.5 ab ± 13.2
20-Aug-13	CH_4_ concentration (ppm)	3.2 a ±1.0	2.8 a ± 0.4	2.7 a ± 0.5	2.8 a ± 0.7
	CO_2_ concentration (ppm)	431.5 a ± 48.3	471.1 a ± 93.1	487.4 a ± 131.1	447.0 a ± 64.2
	N_2_O concentration (ppm)	0.9 a ± 0.4	1.4 a ± 0.6	0.9 a ± 0.4	1.2 a ± 0.3
	CH_4_ FR (g m^-2^ d^-1^)	1.6 a ± 0.5	1.4 a ± 0.2	1.3 a ± 0.2	1.4 a ± 0.4
	CO_2_ FR (g m^-2^ d^-1^)	576.2 a ± 64.4	629.0 a ± 124.3	650.9 a ± 175.1	596.9 a ± 85.8
	N_2_O FR (g m^-2^ d^-1^)	1.3 a ± 0.6	1.9 a ± 0.8	1.2 a ± 0.4	1.7 a ± 0.8
	CH_4_ EF (g AU^-1^ d^-1^)	57.9 a ± 15.3	49.0 a ± 2.6	49.7 a ± 6.0	48.2 a ± 9.0
	CO_2_ EF (kg AU^-1^ d^-1^)	21.2 a ± 1.6	22.1 a ± 1.6	24.3 a ± 4.3	21.1 a ± 1.8
	N_2_O EF (g AU^-1^ d^-1^)	48.5 a ± 25.6	67.3 a ± 31.6	48.5 a ± 18.7	59.1 a ± 28.6
18-Sep-13	CH_4_ concentration (ppm)	3.3 a ±0.3	3.4 a ± 0.7	3.1 a ± 0.5	3.6 a ± 0.7
	CO_2_ concentration (ppm)	389.3 a ± 28.1	381.1 a ± 60.6	423.3 a ± 70.3	422.2 a ± 44.7
	N_2_O concentration (ppm)	0.6 ± 28.1	0.6 ± 60.6	0.6 ± 70.3	0.6 ± 44.7
	CH_4_ FR (g m^-2^ d^-1^)	1.6 a ± 0.1	1.6 a ± 0.4	1.5 a ± 0.3	1.7 a ± 0.4
	CO_2_ FR (g m^-2^ d^-1^)	519.9 a ± 37.5	508.9 a ± 80.9	565.2 a ± 93.9	563.8 a ± 59.7
	N_2_O FR (g m^-2^ d^-1^)	0.8 a ± 0.3	0.7 a ± 0.1	0.7 a ± 0.3	0.8 a ± 0.4
	CH_4_ EF (g AU^-1^ d^-1^)	54.2 a ± 6.0	51.6 a ± 5.7	50.8 a ± 4.0	54.7 a ± 7.7
	CO_2_ EF (kg AU^-1^ d^-1^)	17.3 ab ± 0.6	16.1 b ± 1.1	19.1 a ± 2.1	17.9 ab ± 1.6
	N_2_O EF (g AU^-1^ d^-1^)	26.9 a ± 7.8	24.3 a ± 4.5	24.4 a ± 9.2	23.9 a ± 9.4
9-Oct-13	CH_4_ concentration (ppm)	4.3 a ± 1.4	3.9 a ± 1.1	2.5 a ± 0.2	3.4 a ± 0.2
	CO_2_ concentration (ppm)	367.2 a ± 35.1	381.6 a ± 23.9	345.7 a ± 11.3	379.8 ± 2.9
	N_2_O concentration (ppm)	0.4 a ± 0.0	0.3 a ± 0.1	0.4 a ± 0.1	0.4 a ± 0.0
	CH_4_ FR (g m^-2^ d^-1^)	2.1 a ± 0.7	1.9 a ± 0.5	1.2 a ± 0.1	1.6 a ± 0.1
	CO_2_ FR (g m^-2^ d^-1^)	490.3 a ± 46.9	509.5 a ± 31.9	461.6 a ± 15.0	507.2 a ± 3.9
	N_2_O FR (g m^-2^ d^-1^)	0.5 a ±0.0	0.5 a ± 0.1	0.5 a ± 0.1	0.5 a ± 0.0
	CH_4_ EF (g AU^-1^ d^-1^)	62.2 a ± 16.3	59.1 a ± 15.5	38.8 a ± 1.5	47.8 a ± 5.2
	CO_2_ EF (kg AU^-1^ d^-1^)	14.9 a ± 0.3	15.8 a ± 1.2	15.0 a ± 0.0	14.7 a ± 0.7
	N_2_O EF (g AU^-1^ d^-1^)	14.6 a ± 0.7	14.0 a ± 2.1	15.7 a ± 2.8	13.6 a ± .09

^*^Values followed by the same letter in row are not significantly different at *P* ≤ 0.05; where, *FR* flux rate from pen surface (g m^-2^ d^-1^), *ER* emission rate from pen surface (g hd^-1^ d^-1^)

The effect of fat on gaseous emissions depends on many factors; such as type of fat, amount of fat in feed, and environmental condition. Though the literatures [35, 36] showed that the addition of fat effects enteric CH_4_ production, this study showed that different fat levels from DDGS may not greatly influence the CH_4_ production from the pen surface area. The emissions from the pen surface area are most likely to influence from environmental factors. The environmental conditions were almost similar in all the pen surfaces; therefore, very little variation in gaseous emissions might have been observed under the different treatments conditions. In addition, the reduction of CH_4_ concentration using supplementary fat may not be applicable for corn oil in DDGS; or the application rate of corn oil used in this research may not be sufficient for significant reduction on gaseous emission from pen surface.

When the gaseous emission were compared between different months, significant differences in the gaseous parameters were observed. The CH_4_ emissions were significantly higher during September and October from the pen surfaces as compared to June, July, and August. Higher emissions of CH_4_ were expected due to higher temperatures in July and August [41]. Though the CH_4_ concentration was observed higher in August and July compared to June; the concentration in September and October were even higher than July and August. This could be due to the accumulation of manure on the pen surface that provide anaerobic conditions for CH_4_ emission. Nitrous oxide emissions were significantly lower during September and October and higher during June, July and August (Table 11). The higher temperature during June, July and August could be a reason for higher N_2_O emissions [44]. Similarly, the dry and wet condition of the pen surface due to rain in summer may provide alternate aerobic and anaerobic condition on the pen surface, thus variation of N_2_O emission was observed. The wet conditions of pen surfaces favors anaerobic conditions in manure, resulting in denitrification. Dry conditions favor aerobic conditions in manure resulting in nitrification. Nitrous oxide is produced during both nitrification and denitrification processes [53]. The significantly lowest nitrous oxide and carbon dioxide fluxes during October are most likely due to prevailing dry surface and ambient condition (Table 10).

**Table 11:** Average manure composition on monthly basis

Parameters	June	July	August	September	October
CH_4_ concentration (ppm)	2.2 d^*^ ± 0.0	2.8 c ± 0.2	2.9 bc ± 0.2	3.3 ab ± 0.2	3.5 a ± 0.7
CO_2_ concentration (ppm)	380.2 b ± 5.9	486.2 a ± 19.4	459.3 a ± 21.5	404.0 b ± 19.0	368.6 b ± 14.3
N_2_O concentration (ppm)	1.1 a ± 0.2	1.0 a ± 0.2	1.1 a ± 0.2	0.6 b ± 0.0	0.4 b ± 0.0
CH_4_ FR (g m^-2^d^-1^)	1.1 d ± 0.0	1.4 c ± 0.1	1.4 bc ± 0.1	1.6 ab ± 0.1	1.7 a ± 0.3
CO_2_ FR (g m^-2^d^-1^)	507.6 b ± 7.9	649.2 a ± 26.0	613.3 a ± 21.5	539.4 b ± 19.0	492.1 b ± 14.3
N_2_O FR (g m^-2^d^-1^)	1.4 a ± 0.3	1.4 a ± 0.2	1.5 a ± 0.3	0.8 b ± 0.0	0.5 b ± 0.0
CH_4_ EF (g d^-1^ hd^-1^)	40.5 b ± 1.2	52.7 ab ± 3.8	54.2 b ± 3.8	62.4 a ± 2.3	63.7 a ± 11.6
CO_2_ EF(g d^-1^ hd^-1^)	19487 b ± 624	24958 a ± 326	23584 a ± 326	20693 b ± 143	18541 b ± 431
N_2_O EF (g d^-1^ hd^-1^)	55.2 a ± 11.0	53.3a ± 9.3	58.2 a ± 9.6	29.6b ± 1.4	17.7 b ± 0.9
CH_4_ EF (g d^-1^ AU^-1^)	53.2 ab ± 7.9	57.6 a ± 6.7	51.2 b ± 10.2	52.8 ab ± 6.2	52.0 ab ± 14.9
CO_2_ EF(kg d^-1^ AU^-1^)	25.5 a ± 2.6	26.9 a ± 2.0	22.2 b ± 2.9	17.5 c ± 1.8	15.1 c ± 0.8
N_2_O EF(g d^-1^ AU^-1^)	67.0 a ± 29.0	57.7 a ± 18.5	55.8 a ± 27.7	24.9 b ± 8.1	14.5 c ± 2.0

^*^Values followed by the same letter in row are not significantly different at *P* ≤ 0.05

In comparing the results with the previous study; in 2011, Rahman et al [40] simply measured GHG emission from the same feedlot pen surface and they found that CH_4_, CO_2_ and N_2_O emission were 38, 26, and 17 g hd^-1^d^-1^, respectively, during the 2011 summer period. Similarly, in 2012, Borhan et al. [45] studied the effects of two dietary crude protein levels (12 and 16 %) in the GHG emission on the similar conditions. They found that CH_4_, CO_2_ and N_2_O emission ranged from 40–61, 31–43, and 50–116 gAU^-1^d^-1^ (0.8–1.1, 593–431, and 1–1.9 g m^-2^d^-1^), respectively, during the summer months. They noticed no significant differences on gaseous emission due to different protein diet levels.

Further analsysis was carried out to see the interaction of diet and time on GHG emisison. The results reveal that all CH_4_, CO_2_ and N_2_O emisison (concentration and emission rate) varied significantly (*p* < 0.05) over the sampling period; however, diet did not have any interaction with time for the effect on GHG emissions (Table 12).

**Table 12:** Probability values based on the repeated measure multivariate analysis along with time and treatment interactions

Parameters		Interaction	
	Time		Diet*Time
CH_4_ g m^-2^ d^-1^	<0.01		0.68
CO_2_ g m^-2^ d^-1^	<0.01		0.43
N_2_O g m^-2^ d^-1^	<0.01		0.37
CH_4_ g AU^-1^ d^-1^	0.03		0.41
CO_2_ kg AU^-1^ d^-1^	<0.01		0.97
N_2_O g AU^-1^ d^-1^	<0.01		0.48

### Effect of dietary fat levels on hydrogen sulfide emission

**Fig. 3:**
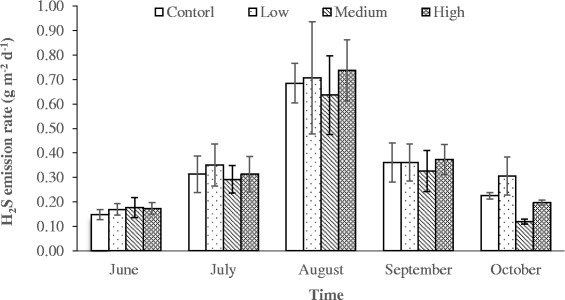
Hydrogen sulfide emission from feedlot pen surfaces in different time period

Hydrogen sulfide concentration was very low (<80 ppb) at the pen surfaces throughout the measurement period. Other researchers have also reported the concentration around 50 ppb in feedlot [54]. There was no significant difference in H_2_S emission rate among dietary treatments. However, variations in H_2_S emission rates were observed during different sampling periods (Fig. 2). The H_2_S emission rate was fairly low (<0.18 g m^-2^ d^-1^) in the first month since pen surfaces had a thin layer of manure on the surface. The H_2_S concentration gradually increased over time and reached up to 0.7 g m^-2^ d^-1^ in August (Fig. 3). However, as the temperature started decreasing (Fig. 2), the H_2_S emission rate also declined gradually (Fig. 3). This study shows that H_2_S emission rate measured on the feedlot pen surfaces were correlated with temperature change (*R*^2^ = 0.49), and manure accumulation (Figs. 2 and 3). Other researchers have also observed very low emission rate of H_2_S from feedlot. Wood et al. [55]) reported the emission rate 103 μg m^-2^ min^-1^. Similarly, Baek et al. [56] and Koziel et al. [57] reported an the H_2_S emission rate as 1.88 μg m^-2^ min^-1^, and 1.39 μg m^-2^ min^-1^, respectively.

## Conclusions

In this study the effect of four dietary fat concentrations (3 to 5.5 % in the composite sample) feed to beef cattle was evaluated in term of manure nutrient composition, VFA concentration, hydrogen sulfide and GHG (CH_4_, CO_2_, and N_2_O) emissions. The study was conducted over a 5-month period from June to October for five ~28-day sampling periods. Overall, the fat levels in the diets showed no or little effect on the manure compositions, VFA, and H_2_S and GHGs emissions. However, some variation in the above mentioned parameters was observed among different measurement periods. In this research, the variation of fat levels from 3 to 5.5 % in cattle diets did not reflect any significant difference on GHGs and H_2_S emission from beef cattle feedlot pen surfaces, as well as on manure composition. It can be concluded that addition of fat to animal diet may not have any impact on gaseous emission and manure compositions.
